# Did hospital mortality in England change from 2005 to 2010? A retrospective cohort analysis

**DOI:** 10.1186/1472-6963-13-216

**Published:** 2013-06-13

**Authors:** Richard M Jacques, James Fotheringham, Michael J Campbell, Jon Nicholl

**Affiliations:** 1 School of Health and Related Research (ScHARR), University of Sheffield, 30 Regent Street, Sheffield S1 4DA, UK

**Keywords:** Hospital mortality, Quality indicators, Case mix adjustment, Trends, Retrospective study

## Abstract

**Background:**

There is some evidence that hospital performance in England measured by the Dr Foster Hospital Standardised Mortality Ratio (HSMR) has improved substantially over the last 10 years. This study explores mortality in-hospital and up to 30 days post-discharge over a five year period to determine whether there have been improvements in case-mix adjusted mortality, to examine if any changes are due to changes in case-mix adjustment variables such as age, sex, method of admission and comorbidity, and to compare changes between hospital trusts.

**Methods:**

Using Hospital Episode Statistics linked to mortality data from the Office for National Statistics the Summary Hospital-Level Mortality Index (SHMI) was calculated for all patients who were discharged or died in general acute hospital trusts in England for the period 01/04/2005 to 30/09/2010.

**Results:**

During this five year period the number of admissions rose by 8% but deaths fell by 5%. The SHMI fell by 24% from 112 to 85 over the period, partly due to fewer deaths but partly due to increasing numbers predicted by the SHMI model. Excluding comorbidities from the model the SHMI fell by 18% from 108 to 89 over this period. The reduction was similar in emergency and elective admissions and in all other sub-groups examined. The average quarterly change in SHMI varied considerably between trusts (range: -4.4 to −0.2).

**Conclusions:**

As measured by the SHMI there has been a 24% improvement in mortality in acute general trusts in England over a period of five and a half years. Part of this improvement is an artificial effect caused by changes in the depth of coding of comorbidities and other effects due to change in case-mix or non-constant risk.

## Background

There is some evidence that hospital performance in England has improved substantially over the last 10 years. The Dr Foster Hospital Standardised Mortality Ratio (HSMR) for admissions to all English hospitals taken together between 2000 and 2010 fell by 42% from 115 in 2002 to 67 in 2011 [1], and there was a 21% reduction in an age, sex and diagnosis adjusted HSMR for emergency admissions between 2004/5 and 2008/9 [2]. This could be interpreted as a result of large gains in the quality of care in recent years. However, there are doubts about whether HSMRs are closely related to quality of care [3], or can ever be used to reliably measure quality of care [4]. Empirical studies have also demonstrated that different methods of calculating standardised mortality measures rank the performance of hospitals differently [5,6], raising doubts about their reliability. The influence of comorbidity on risk of death has been show to vary across hospitals, including greater burden of comorbidity actually being protective [7]. Although there is evidence of the constant risk fallacy, exclusion of comorbidity has been shown to change the HSMR by more than 5 points in only 16% of trusts [6]. Local organisation of care and disease severity remains uncaptured. Nevertheless, a fall of 30% in hospital mortality, even if it is only true in part, would point to important medical advances and/or better hospital care. It would help justify the 34% increase in NHS hospital expenditure over the same period [8], and might suggest that some of the many initiatives affecting practice, policy, and the organisation of the NHS had had some benefits.

Because the Dr Foster HSMR only focuses on in-hospital deaths for patients admitted with a subset of conditions, and in response to methodological concerns and transparency issues [9] a new measure - the Summary Hospital-Level Mortality Index (SHMI) – was developed. The SHMI includes all admissions and includes all deaths up to 30 days post discharge in order to avoid potential biases in using in-hospital deaths [10]. Using the SHMI we have therefore sought to validate the Dr Foster finding, investigated if any changes in the national SHMI are due to changes in case-mix adjustment variables such as age, sex, method of admission and comorbidity, and compared changes between hospital trusts.

## Methods

### Data

We were supplied with a dataset by the NHS Information Centre for the purpose of statistical modelling of the new SHMI indicator, including the impact of case-mix adjustment variables and the variability of the measure over time. The dataset comprised of all admissions to English hospitals from the Hospital Episode Statistics (HES) data warehouse for spells which ended between 01/04/2005 and 30/09/2010. Date of death data supplied by the Office for National Statistics (ONS) was linked to the hospital episode data set and deaths within 30 days of discharge were identified. All patient data provided was anonymised prior to receipt by the authors.

We followed the previously described methodology for processing the linked hospital episode data before calculating the SHMI [11,12]. Briefly, this involved excluding maternity admissions, day case admissions, and admissions to private and community hospitals. We also excluded admissions to 72 Specialist trusts. There was no formal definition of General/Specialist status and we took the definition of general trusts from lists reported by other mortality indicator providers.

Categories were created for all variables. Age was split into 5 year age bands except for infants aged 0–1 and preschool children aged 1–4. A comorbidity score was derived by converting secondary diagnosis codes into the 19 clinical conditions identified in the Charlson comorbidity index [13], with contemporary weights for the presence of individual conditions contribution to the overall score [14]. The Index of Multiple Deprivation rank (an area level deprivation measure derived from the patient’s postcode) was reported by HES and grouped by fifths. Type of admission was grouped into emergency and elective.

The reason for admission was identified from the ICD10 code in the first diagnosis field, and collapsed into the diagnostic groups given by the Agency for Healthcare Research and Quality [15]. Diagnosis groups were then combined into the 138 groups used in the calculation of the SHMI [12]. It has previously been reported that the mean c statistic over all diagnosis groups in the SHMI model was 0.830 (range 0.534 – 0.970) and that the coefficient of determination R^2^ showed the SHMI model accounted for 81% over the total variability [12].

### Statistical methods

We estimated the probability of death in hospital or within 30 days of discharge for all completed admissions for the period 01/04/2005 to 30/09/2010 by fitting logistic regression models using the SHMI covariates (age, sex, method of admission and comorbidity) within diagnosis group. We accounted for the effect of seasonal variation in hospital admissions by including an extra categorical variable for month of admission in each of the logistic regression models. We then summed these probabilities predicted by the model over all diagnosis groups and for each trust for each consecutive 3 month period to obtain the expected number of deaths per trust for each quarter. The ratio of the observed number in each quarter to the expected is equivalent to indirect standardisation [16]. Fitting one model to the data from all five years combined means that we can make valid comparisons over time. This is because we calculate one set of case-mix weights for all time periods instead of the weights changing over time (which would be the case if separate models were fitted for each year or quarter).

We plotted the quarterly values of the SHMI, expected number of deaths and observed number deaths in all hospitals against time for the five year period. Coding levels of the comorbidity variable have changed over this time period so we examined the effect of removing comorbidity from the model so that we could be sure any trends identified were not a result of these changes. Further analyses examined the quarterly changes in SHMI in subgroups of age, sex, admission method, index of deprivation and comorbidity. As the SHMI model adjusts for age, sex, admission method and comorbidity we would not expect to see differences in the overall SHMI between the subgroups. However, trend is not adjusted for in the SHMI model so we can investigate any differences between subgroups in terms of their time trend.

We estimated the linear trend in individual hospital SHMIs by ordinary least squares regression of the 22 quarterly SHMIs on time. The regression coefficients were plotted on a funnel plot with control lines calculated in a conventional manner [17,18], Winsorising the 20% most extreme values to examine whether there were any extremes in the rate of change.

## Results

**Figure 1:**
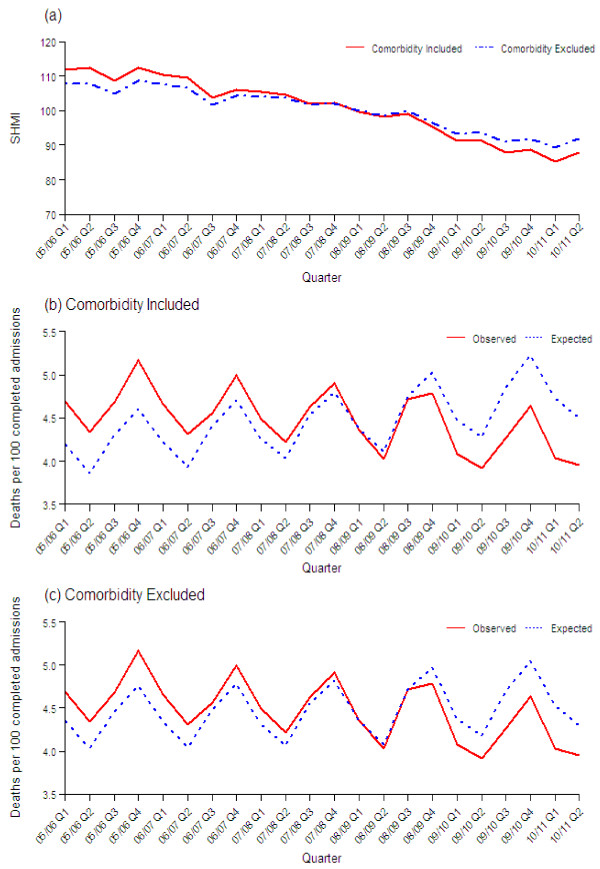
Quarterly values of (a) SHMI with and without comorbidity included in the model, and observed and expected number of deaths per 100 completed admissions when (b) comobidity is included in the model, and (c) when comorbidity is excluded from the model.

Over the five and a half year period there has been an increase of 8% in the total number of admissions per year meeting our inclusion criteria, but a fall of 5% in the number of deaths in-hospital or up to 30 days post discharge (Table 1). Adjusting for changing case-mix, the SHMI fell by 24% from 112 in the first quarter of 2005/06 to 85 in the first quarter of 2010/11 (Figure 1a). This reduction occurred both as a result of a fall in the observed death rate and an increase in the expected death rate (Figure 1b). Excluding comorbidity from the model the SHMI fell by 18% from 108 in the first quarter of 2005/06 to 89 in the first quarter of 2010/11 (Figure 1a). This reduction also occurred as a result of a fall in the observed death rate and an increase in the expected death rate (Figure 1c).

**Table 1:** Summary statistics of completed admissions (England 01/04/2005 to 30/09/2010)

**Year**	**2005/6**	**2006/7**	**2007/8**	**2008/9**	**2009/10**	**2010/11**^**‡**^
**Number of Completed Admissions**	6127090	6117270	6179654	6430732	6533295	3284595
**Deaths**	289288	283407	281890	287755	276185	131236
	(4.72)	(4.63)	(4.56)	(4.47)	(4.22)	(4.00)
**Age**						
Mean	50.5	50.7	50.6	51.0	51.2	51.6
≥75	1481427	1490421	1512603	1613590	1660950	844161
	(24.18)	(24.36)	(24.48)	(25.09)	(25.42)	(25.70)
**Sex**						
Male	3067508	3078058	3106639	3225150	3272829	1645890
	(50.06)	(50.32)	(50.27)	(50.15)	(50.09)	(50.11)
Female	3058803	3038271	3071973	3203801	3260081	1638502
	(49.92)	(49.67)	(49.71)	(49.82)	(49.90)	(49.88)
Missing	779	941	1042	1781	385	203
	(0.01)	(0.01)	(0.02)	(0.03)	(0.01)	(0.01)
**Admission Method**						
Emergency	4583900	4620067	4688529	4939154	5092303	2571408
	(74.81)	(75.52)	(75.87)	(76.81)	(77.94)	(78.29)
Elective	1534313	1494969	1488747	1489467	1439186	712293
	(25.04)	(24.44)	(24.09)	(23.16)	(22.03)	(21.69)
Missing	8877	2234	2378	2111	1806	894
	(0.14)	(0.04)	(0.04)	(0.03)	(0.03)	(0.02)
**Comorbidity Group**						
0	4561016	4446238	4412397	4484075	4391532	2136832
	(74.44)	(72.68)	(71.40)	(69.73)	(67.21)	(65.06)
1-5	721762	766629	794970	862211	932887	491909
	(11.78)	(12.53)	(12.86)	(13.41)	(14.28)	(14.97)
>5	844312	904403	972287	1084446	1208876	655854
	(13.78)	(14.78)	(15.73)	(16.86)	(18.50)	(19.97)

Values are N (%) unless otherwise stated.

^‡^Values for 2010/11 are based on the first two quarters only.

**Figure 2:**
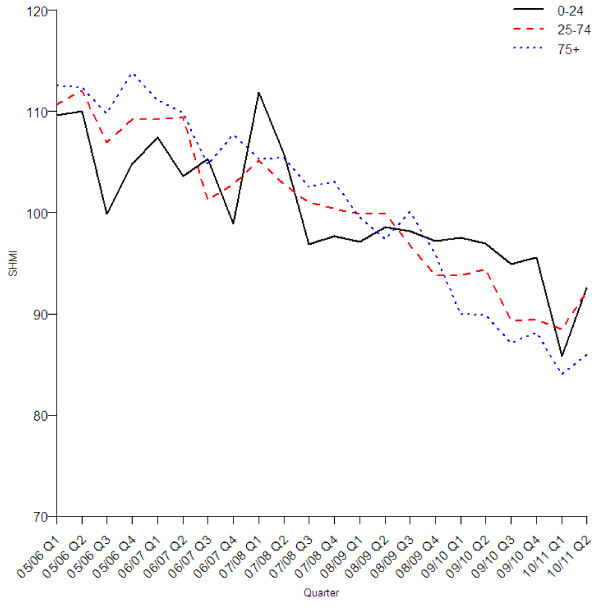
Quarterly values of SHMI by age group.

**Figure 3:**
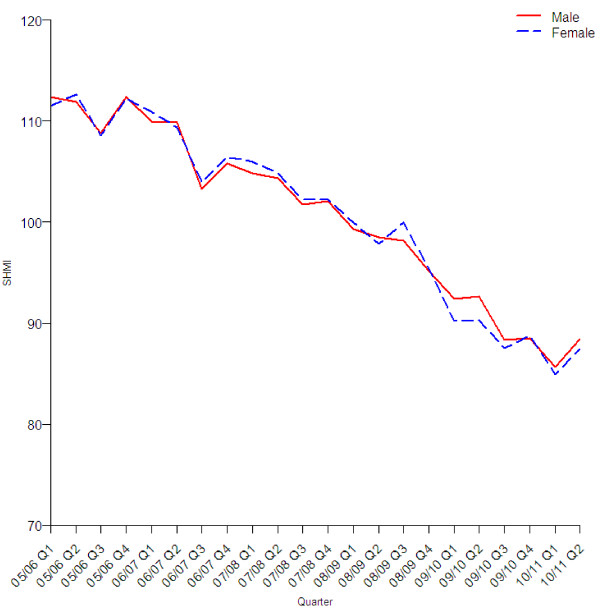
Quarterly values of SHMI by sex.

**Figure 4:**
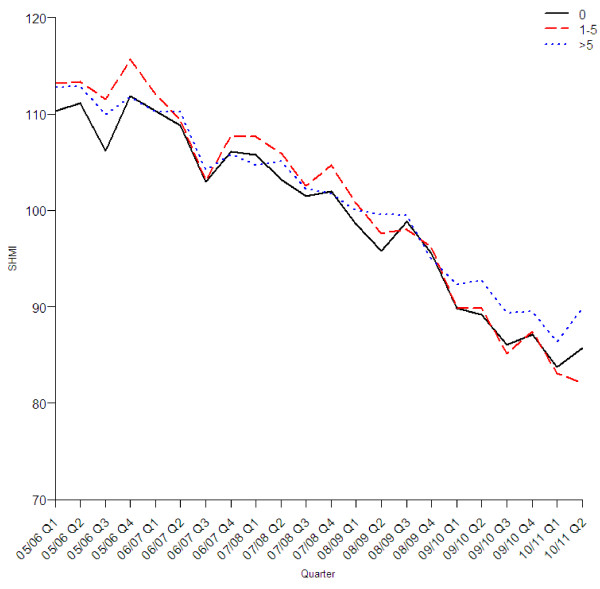
Quarterly values of SHMI by comorbidity group.

**Figure 5:**
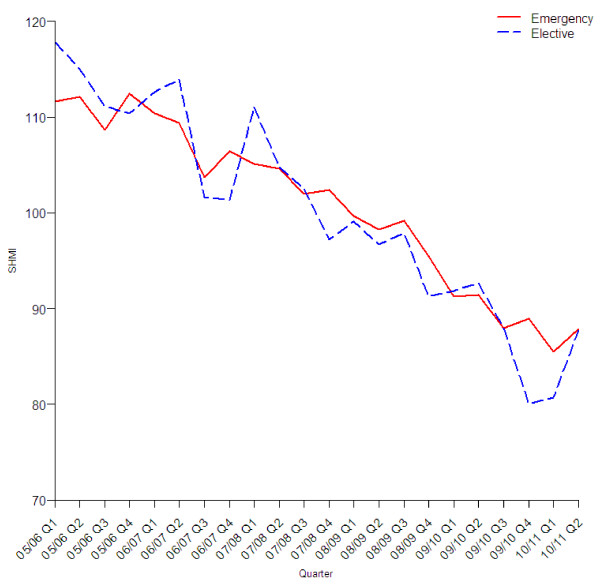
Quarterly values of SHMI by method of admission.

**Figure 6:**
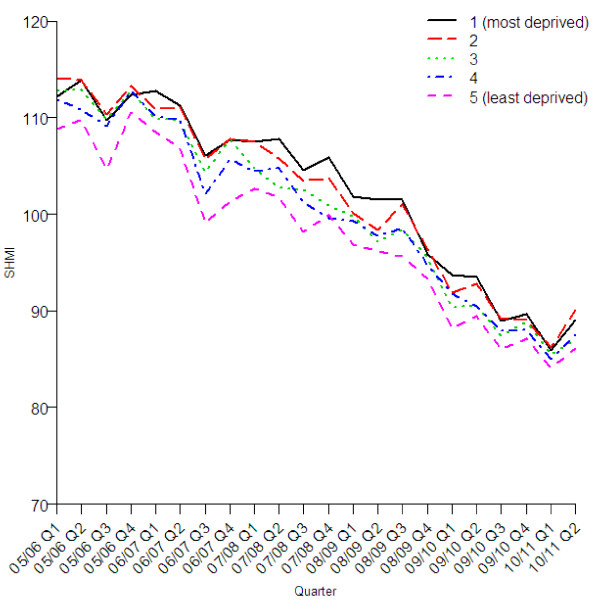
Quarterly values of SHMI by fifth of index of multiple deprivations.

The reduction in the standardised mortality rate was similar in all age groups (Figure 2), sexes (Figure 3), three groups with different levels of recorded comorbidity (Figure 4), elective and emergency admissions (Figure 5), and patients from areas of different levels of deprivation (Figure 6).

Figure 5 shows that the SHMI for elective admissions is much more variable from quarter to quarter than that of emergency admissions. This variation appears to be seasonal with a reduction in the SHMI for elective admissions in the fourth quarter of each reporting year, that is January to March. Figure 6 shows the subgroups of fifths of the index of multiple deprivation, the trend is similar for each fifth. The expected probabilities from the SHMI model are not adjusted for deprivation as this was not found to significantly improve the discrimination between hospitals [11,12].

**Figure 7:**
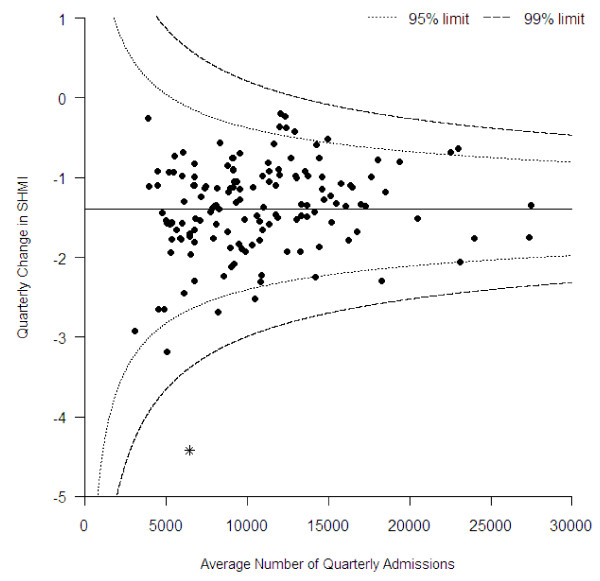
Funnel plot showing the average number of quarterly admissions and average quarterly change in SHMI: *, Mid Staffordshire Trust.

The estimated quarterly reduction in the SHMI varied considerably between hospitals from a maximum reduction of −4.4 points per quarter to a minimum of −0.2 points per quarter. Plotting these on a funnel plot shows that all trusts are within the 99.9% limits with the exception of the Mid Staffordshire Trust which is outlying with a large average quarterly decrease (Figure 7).

## Discussion

### Do the changes really indicate improving quality of care?

We have found a decline in the SHMI of 24% over the 5 year period. We have previously suggested that effects like this should be put to a number of tests before they are accepted as indicating real changes in performance [12]. These tests include:

i. Is any change in the SHMI the result of a change in the observed death rate or the expected death rate?

ii. Is a difference in the SHMI sensitive to the methods used? For example, is it sensitive to how the standardisation is carried out or the weightings used?

iii. Is there any corroborating evidence from related quality of care indicators?

Determining all of the individual factors that have influenced the change in SHMI would be extremely challenging. More broadly, we have looked at changes in the observed death rate and found that deaths up to 30-days post discharge have fallen by 15% from 4.7 to 4.0 per 100 admissions over this 5 year period. Explanations include improved clinical care, more deaths in the community without accessing secondary services and improving population health.

The number of expected deaths has increased by 15% from 3.9 per 100 admissions in Q2 2005/06 to 4.5 per 100 admissions in Q2 2010/11. Changes in SHMI variables that drive the increase in expected deaths include a small increase in the average age of patients (50.5 to 51.6), an increase in the proportion admitted as emergencies (75% to 78%), and a large increase in the proportion of patients recorded with comorbidities (26% to 35%) (see Table 1), all of which are assigned greater risk of death in the SHMI model. The 15% fall in the observed death rate is amplified by the increasing age of patients and the increase in the proportion of patients admitted as emergencies, patient groups more likely to die than their younger elective counterparts. Whilst the changes in age and method of admission may reflect the characteristics of the population or admission policy/thresholds, the change in comorbidities may just reflect a change in coding practice.

Population age should only increase the expected number of deaths if the age-specific risk is constant over time. Indirect standardisation models used to produce standardised mortality rates (SMRs) like the SHMI assume that the risk associated with a risk factor such as age is constant between places and over time [19]. So, for example, the model assumes that the risk associated with a particular age is the same at the beginning of the five year period as at the end. Population mortality rates have improved by 10% over the same period [20] suggesting that, due to improving population health, the risk at a particular age is declining and this will result in a fall in SHMI.

An increase in the number of admissions coded as emergency over this period has been reported elsewhere as a result of a growth in admissions lasting a day or less, and predominantly in people aged 25 to 60 years of age [2]. A likely explanation is that some emergencies previously managed out of hospital are being admitted, leading to the growth of short length of stay admissions. It is possible therefore that the reduction in the SHMI is due to an increase in less severe cases who are more likely to survive. This along with the concurrent decrease in elective admission mortality and improvements in all bands of comorbidity (Figures 4 and 5) suggests a difference in admission case-mix is not responsible for improvements in the SHMI.

Our finding that the model without comorbidities found an estimated annual change in the SHMI of −3.6 compared to −4.9 with comorbidities, indicates that changes in coding of comorbidities do not explain the majority of the reduction in the SHMI over this 5 year period. The change in comorbidity over this period may reflect a genuine increase in underlying comorbidity in admitted patients but it more likely reflects an improvement in the hospital’s capacity to record underlying comorbidity.

It looks therefore as if part of the improvement in the SHMI is due to a reduction in the numbers and rate of death brought about by improvements in care; part is an artificial effect caused by changes in the coded comorbidities over time; and the remainder may be due to other real or artificial effects due to changes in case-mix or non-constant risk.

There is some corroborating evidence that there have been real improvements in care from more detailed audits of outcomes in specific clinical conditions such as acute myocardial infarction and stroke [21], chronic obstructive pulmonary disease [22], head injury [23], and hip fracture [24], which have found a fall in in-hospital mortality during this period. These reductions have been ascribed to improvements in care brought about for many reasons such as advances in medical technologies and the introduction and implementation of evidence-based guidelines. It should also be remembered that during this period NHS net expenditure in England increased by 34% to 99.8bn pa [8], increased competition between hospitals was created, payment by results introduced, and a number of programmes focusing specifically on quality and safety of hospital care introduced which has resulted for example in a 64% reduction in C. difficile and a 78% reduction in MRSA reported infections in hospitals over this period [25].

A more direct comparison with a mortality measure such as the Dr Foster HSMR was not performed as publically available data are recalibrated annually and would mask changes in the expected death rate over time. Theoretically the SHMI should be more robust to changes in discharge and community care policy than the HSMR as it incorporates death at 30 days from discharge.

### Variation between hospitals

We have also examined variation between hospitals in this trend. The results show that improvements have been widespread but there are some hospitals where almost no improvement has been seen and others where large improvements have been recorded. One hospital has shown an exceptional improvement and that is the Mid-Staffordshire Hospital Trust which reduced its SHMI at about 4.4 percentage points each quarter and was well outside the 99.9% control limit. Whilst the SHMI is described as being used by the DH to *monitor* hospital performance [9], in reality because the weights are recalculated every quarter, the expected values change and it is actually only being used to *compare* hospital performance. We think that the Department of Health should monitor trends in order to identify any hospitals where the SHMI is going in the wrong direction, or changing their coding practice so that hospital comparisons are unreliable. We don’t think this needs analysis over a five year period as we have done. A sensible approach would be for a rolling analysis which compared two consecutive years using funnel plots to see year on year differences between hospitals.

## Conclusions

There has been a 24% improvement in mortality in acute general trusts in England over a period of five and a half years as measured by the SHMI. This improvement is due to a decrease in the number of observed deaths and an increase in the number of expected deaths. The reduction in the number of observed deaths is in part due to falling mortality rates in the general population, but may also be in part due to improvements in hospital care. The increase in the expected number of deaths is partially an artificial effect due changes in the amount of coded comorbidities; and the remainder may be due to other changes in case-mix or non-constant risk. However, there is some evidence that hospital mortality has improved over this five year period.

## Abbreviations

HES: Hospital episode statistics; HSMR: Hospital standardised mortality ratio; ICD: International classification of diseases; NHS: National Health Service; ONS: Office for National Statistics; SHMI: Summary hospital-level mortality index.

## Competing interests

The authors declare that they have no competing interests.

## Authors’ contributions

All authors designed the study. RMJ and JF carried out the statistical analysis. JN wrote the first draft of the manuscript. All authors were involved in editing consecutive drafts of the manuscript, interpreted the findings, and approved the final draft.

## Pre-publication history

The pre-publication history for this paper can be accessed here:

http://www.biomedcentral.com/1472-6963/13/216/prepub
